# Point-of-care Ultrasonography for Detecting the Etiology of Unexplained Acute Respiratory and Chest Complaints in the Emergency Department: A Prospective Analysis

**DOI:** 10.7759/cureus.3218

**Published:** 2018-08-28

**Authors:** Layton Lamsam, Laleh Gharahbaghian, Viveta Lobo

**Affiliations:** 1 School of Medicine, Stanford University, Stanford, USA; 2 Department of Emergency Medicine, Stanford University School of Medicine, Stanford, USA

**Keywords:** chest pain, radius, dyspnea, point-of-care ultrasound, bedside ultrasound, ultrasound, chest radiography, chest ct, emergency department, rapid assessment of dyspnea in ultrasound

## Abstract

Introduction

Point-of-care ultrasound (POCUS) is increasingly used as a diagnostic tool in emergency departments. As the number and type of POCUS protocols expand, there is a need to validate their efficacy in comparison with current diagnostic standards. This study compares POCUS to chest radiography in patients with undifferentiated respiratory or chest complaints.

Methods

A prospective convenience sample of 59 adult patients were enrolled from those presenting with unexplained acute respiratory or chest complaints (and having orders for chest radiography) to a single emergency department in an academic tertiary-care hospital. After a brief educational session, a medical student, blinded to chest radiograph results, performed and interpreted images from the modified Rapid Assessment of Dyspnea in Ultrasound (RADiUS) protocol. The images were reviewed by a blinded ultrasound fellowship-trained emergency physician and compared to chest radiography upon chart review. The primary “gold standard” endpoint diagnosis was the diagnosis at discharge. A secondary analysis was performed using the chest computed tomography (CT) diagnosis as the endpoint diagnosis in the subset of patients with chest CTs.

Results

When using diagnosis at discharge as the endpoint diagnosis, the modified RADiUS protocol had a higher sensitivity (79% vs. 67%) and lower specificity (71% vs. 83%) than chest radiography. When using chest CT diagnosis as the endpoint diagnosis (in the subset of patients with chest CTs), the modified RADiUS protocol had a higher sensitivity (76% vs. 65%) and lower specificity (71% vs. 100%) than chest radiography. The medical student performed and interpreted the 59 POCUS scans with 92% accuracy.

Conclusion

The sensitivity and specificity of POCUS using the modified RADiUS protocol was not significantly different than chest radiography. In addition, a medical student was able to perform the protocol and interpret scans with a high level of accuracy. POCUS has potential value for diagnosing the etiology of undifferentiated acute respiratory and chest complaints in adult patients presenting to the emergency department, but larger clinical validation studies are required.

## Introduction

Chest radiography is the imaging modality of choice for the initial diagnostic evaluation of the undifferentiated patient with a respiratory or chest complaint. Unfortunately, chest radiograph has a sensitivity of 47% for the detection of pleural effusion, 52% for pneumothorax, 77% for pneumonia, and 69.5% for pulmonary edema [1-4]. These findings, along with the time required to obtain a chest radiograph, make it a suboptimal imaging modality for time-sensitive resuscitation needs in the critically ill.

Point-of-care ultrasound (POCUS) is increasingly used due to the ability to be quickly performed and interpreted at the bedside by a physician and the lack of radiation exposure. A multitude of studies have confirmed the efficacy of POCUS in the dyspneic patient, with various protocols in existence [5-8]. Lichtenstein and Mezière published the Bedside Lung Ultrasound in Emergency (BLUE) protocol to screen dyspneic patients for acute respiratory failure (ARF) and found it to be 90.5% accurate in determining the etiology of ARF [5]. In addition, Zanobetti et al. compared POCUS and chest radiography in acutely dyspneic patients and found that ultrasound had a higher sensitivity than chest radiography for detecting pleural effusion and lung consolidation [6].

However, the Rapid Assessment of Dyspnea with Ultrasound (RADiUS) protocol was the first to standardize the POCUS approach to undifferentiated dyspneic patients [7]. The RADiUS examination involves four components. These include (1) a focused cardiac examination for pericardial effusion, left ventricular dysfunction, and right ventricular dilation (indicating right ventricular strain), (2) a focused inferior vena cava (IVC) evaluation to estimate intravascular volume status, (3) evaluation of the thoracic cavity for pleural effusions, and (4) evaluation of the pleural line for pneumothorax and interstitial fluid.

The primary goal of this study was to compare a modified version of the RADiUS protocol with chest radiography in patients presenting to the emergency department (ED) with an undifferentiated respiratory or chest complaint. This comparison was made using two different endpoint, or “gold standard”, diagnoses: (1) clinical diagnosis at discharge and (2) chest computed tomography (CT) diagnosis. The secondary goal was to determine if it was feasible for a medical student to learn and use the modified RADiUS protocol in a clinical setting.

## Materials and methods

**Figure 1:**
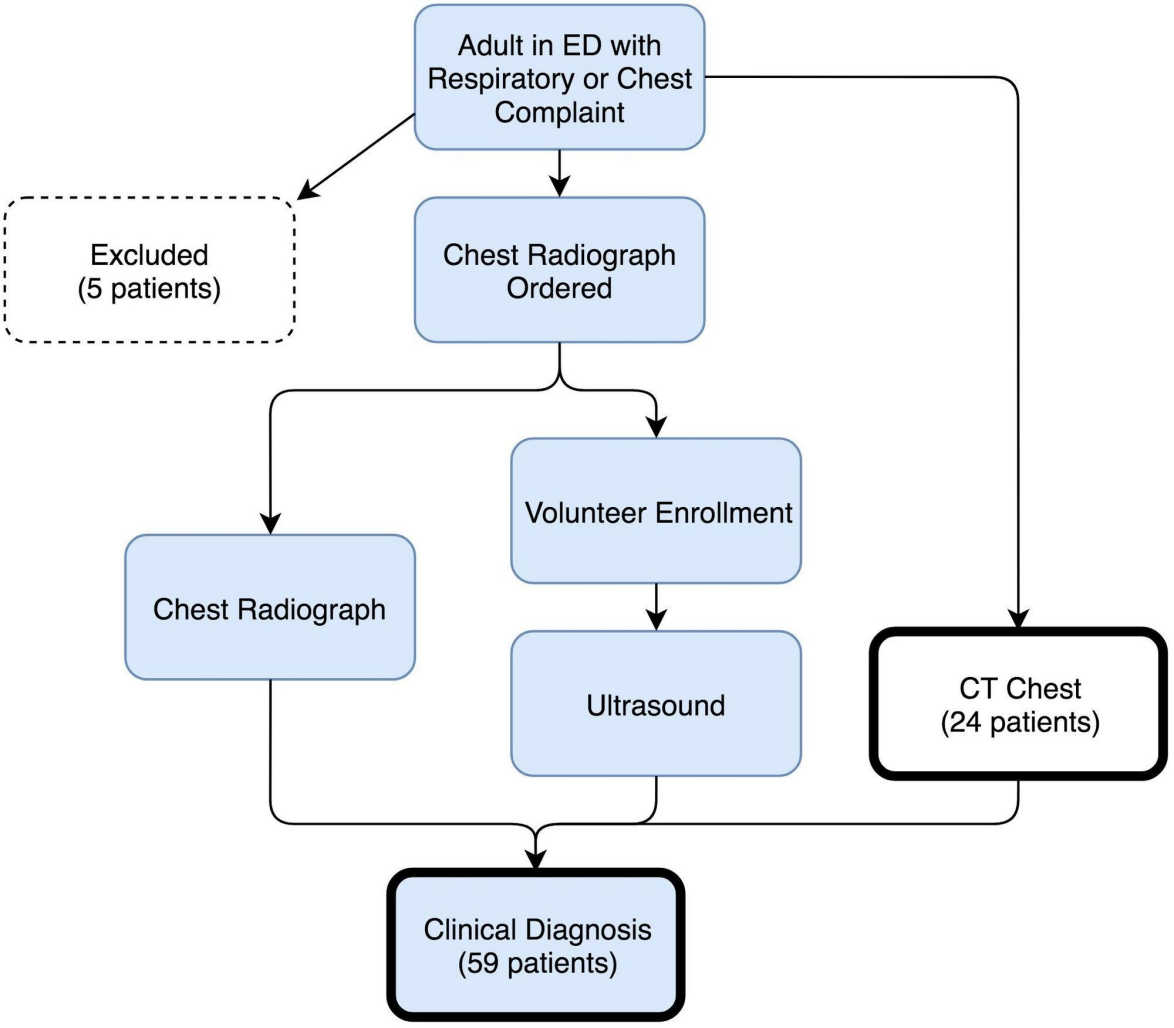
Study Flow Diagram Blue fill color indicates steps required for inclusion, and bold outlines indicate diagnostic endpoints. (ED = Emergency Department, CT = Computed Tomography)

A convenience sample of adult patients (aged at least 18 years) presenting to the emergency department over a two-month period with a respiratory or chest chief complaint and who had an order for a chest radiograph (either 1-view or 2-view) as part of their initial evaluation were included in the study (Figure 1).

A medical student reviewed a log of active patients in the emergency department. Patients who fit the inclusion criteria were approached for enrollment after obtaining the consent of the primary ED team. The student did not know any additional history, physical exam findings, or laboratory and imaging results. All POCUS scans were performed after an initial two-hour training session with an ultrasound fellowship-trained emergency physician who observed the student perform and interpret 15 modified RADiUS ultrasound protocols correctly. The 10-5 MHz linear array and 5-1 MHz phased array probes on a SonoSite M-Turbo® or Edge® system (FUJIFILM SonoSite, Inc., WA, USA) were used to complete the various POCUS applications that are a part of the modified RADiUS protocol (Table 1).

**Table 1:** Modified RADiUS Protocol All components use the phased array probe unless otherwise stated. Modifications to the RADiUS protocol are italicized. (POCUS = point-of-care ultrasound, RADiUS = Rapid Assessment of Dyspnea with Ultrasound, IVC = inferior vena cava).

Protocol Component	Technique
Cardiac examination	Perform with the patient at a 45-degree angle when possible. Include the parasternal long-axis, parasternal short-axis, apical four-chamber, and subxiphoid views. Estimate contractility, chamber size, and any pericardial fluid.
IVC evaluation	Perform at the subxiphoid view and measure the diameter and percent change with respiration. The right lateral view may be used if the IVC cannot be visualized at the subxiphoid view.
Thoracic cavity evaluation	Assess the costophrenic angles for pleural effusion.
Pleural line assessment	Assess second intercostal spaces for lung sliding using the linear probe. Assess each of the eight Volpicelli lung zones for B-lines, A-lines, and comet tails.

The RADiUS protocol does not provide a standardized set of ultrasound markers for the identification of clinical patterns, so a literature review was performed to establish sets of ultrasound markers for 11 clinical patterns (Table 2). The RADiUS protocol was modified to better assess for these 11 clinical patterns; the parasternal short-axis view was added to the focused cardiac examination. This view was included to maximize the ability of the student to assess ventricular contractility and the D-shaped sign [9]. In addition, when unable to perform an IVC evaluation using the RADiUS subxiphoid view, a right lateral view was used. Finally, assessment of the eight Volpicelli lung zones was included to aid in the detection of focal interstitial disease, pulmonary edema, and pneumonia [10].

**Table 2:** Ultrasound Clinical Patterns Each clinical pattern corresponds to ultrasound markers from the modified RADiUS protocol. (POCUS = point-of-care ultrasound, mirror-image artifact [11], spine sign [12], B-lines [13], comet tails [14], lung point [15], hepatization of lung [16], A lines [13], D-shaped sign [9]).

Ultrasound Clinical Patterns	Ultrasound Markers
Pleural effusion	Anechoic areas above diaphragm viewed in the dependent chest areas, loss of mirror-image artifact, positive spine sign
Focal interstitial disease [Right or Left]	Two or more B-lines in one or more (but not all) regions of a lung field
Pulmonary Edema	More than two B-lines in more than two lung regions, bilaterally
Pneumothorax [Right or Left]	Absence of lung sliding and comet tails, presence of lung point
Pneumonia	Focal hypoechogenic areas with B lines, variation in lung sliding, hepatization of lung with air bronchograms
Pulmonary fibrosis pattern	Multiple comet tails (more than eight per field) with thickened and irregular pleural line
Chronic obstructive pulmonary disease (COPD)	Multiple bilateral A lines without evidence of other pathology
Pericardial effusion	Circumferential anechoic area within pericardium in one or more cardiac views
Cardiac tamponade	Pericardial effusion with right ventricle collapse during diastole
Systolic heart failure	Reduced contractility of left ventricle,inferior vena cava (IVC) diameter over 2.5 cm with less than 50% change with respiration
Pulmonary embolism	Right ventricular enlargement with septal bowing towards Left ventricle (D-shaped sign)
No emergent pathology	No markers

All included scans were conducted and interpreted by the medical student before being archived, and radiology was blinded to POCUS results. The presence of pathology corresponding to any of the 11 clinical patterns was recorded. The scans were later reviewed by an ultrasound fellowship-trained emergency medicine physician who was blinded to the medical student’s interpretation. Subsequently, a chart review was performed to obtain the final radiology interpretation of the chest radiography and CT chest and to obtain the diagnoses at discharge from the ED (or from the hospital if the patient was admitted). This study was approved by the hospital institutional review board prior to the start of data collection.

R Version 3.1.3 (R Project, GNU) and the DTComPair package (authors: Christian Stock, Thomas Hielscher) were used in the data analysis [17, 18]. Diagnoses were collapsed into binary variables: clinical and chest CT diagnoses were set to 1 if one or more of the detectable pathologies (indicated by clinical patterns) were present, and 0 otherwise. Using the clinical diagnosis endpoint, ultrasound and chest radiograph diagnoses were set to 1 if they diagnosed all of the detectable pathologies noted by the clinical diagnosis, and 0 otherwise. Results were coded likewise when compared to chest CT.

Statistical significance was defined as a p-value less than 0.05. The sensitivity, specificity, positive predictive value, and negative predictive value were calculated for POCUS and chest radiography twice, using discharge diagnosis as the primary diagnostic endpoint and chest CT diagnosis as the secondary diagnostic endpoint [19]. The p-values between sensitivities and specificities were calculated using McNemar’s test [20]. The p-values between positive predictive values and negative predictive values were calculated using a weighted generalized score statistic [21].

## Results

Fifty-nine patients were included in the study with an average age of 59.5 years and 57.6% being male. All parts of the RADiUS exam were performed on all patients. Five patients were excluded: three did not receive chest radiographs, one did not have a respiratory or chest chief complaint, and one did not have a clinical diagnosis. The two most common chief complaints at triage were chest pain and dyspnea, and 49.2% of all patients were admitted to the hospital (Table 3). Out of 59 patients, 24 received a chest CT. Review of the ultrasound images by an ultrasound fellowship-trained emergency physician revealed four patients that the student classified as having no emergent pathology, but who did have ultrasound clinical patterns. In addition, there were two patients for whom the student found only some of the ultrasound clinical patterns that were present. Overall, the medical student correctly interpreted 54 out of 59 patient scans (92%).

**Table 3:** Cohort Demographics

Demographic	Mean / Percent
Age	59.5 y
Male	57.6 %
Admitted	49.2 %
Chief complaint: dyspnea	30.5 %
Chief complaint: chest pain	49.2 %
Chief complaint: other	20.3 %

Using clinical diagnosis as the diagnostic endpoint, POCUS had higher sensitivity (79% v. 67%, p = 0.37) and lower specificity (71% v. 83%, p = 0.16) than chest radiography. POCUS also had lower positive (66% v. 73%, p = 0.41) but higher negative (83% v. 78%, p = 0.55) predictive values than chest radiography (Table 4). Using chest CT diagnosis as the diagnostic endpoint, POCUS had a higher sensitivity (76% v. 65%, p = 0.41) and lower specificity (71% v. 100%, p = 0.16) compared to chest radiography. POCUS also had lower positive (87% v. 100%, p = 0.19) and higher negative (56% v. 54%, p = 0.90) predictive values than chest radiography (Table 5).

**Table 4:** Comparison with Clinical Diagnosis as the Diagnostic Endpoint (POCUS = point-of-care ultrasound, CXR = chest radiography, PPV = positive predictive value, NPV = negative predictive value).

Statistic	POCUS (95% CI)	CXR (95% CI)	p-value
Sensitivity	79% (63 – 95)	67% (48 – 86)	0.37
Specificity	71% (56 – 86)	83% (70 – 95)	0.16
PPV	66% (48 – 83)	73% (54 – 91)	0.41
NPV	83% (70 – 97)	78% (65 – 92)	0.55

**Table 5:** Comparison with Chest CT Diagnosis as the Diagnostic Endpoint (CT = computed tomography, POCUS = point-of-care ultrasound, CXR = chest radiography, PPV = positive predictive value, NPV = negative predictive value).

Statistic	POCUS (95% CI)	CXR (95% CI)	p-value
Sensitivity	76% (56 – 97)	65% (42 – 87)	0.41
Specificity	71% (38 – 100)	100% (NA)	0.16
PPV	87% (69 – 100)	100% (NA)	0.19
NPV	56% (23 – 88)	54% (27 – 81)	0.90

## Discussion

This study is the first to compare the modified RADiUS protocol with chest radiography in patients with unexplained respiratory or chest complaints. Using either the primary endpoint of clinical diagnosis at discharge or the secondary endpoint of chest CT imaging, there was no significant difference in sensitivity or specificity between POCUS and chest radiography. This study offers advantages over the two related studies: first, while Lichtenstein and Mezière standardized their ultrasound protocol, the scope was limited to acute respiratory failure [5]; second, Zanobetti et al. captured a wider range of diagnoses, but they were unable to calculate sensitivity and specificity values outside of those patients receiving CT scans [6]. These positive initial results are an encouraging indication that the RADiUS protocol and its variants merit additional investigation.

Multicenter studies and meta-analyses have shown results consistent with the many single-site studies that have demonstrated POCUS to be a valuable diagnostic tool in various emergent settings. A systematic review published in 2010 found that ultrasound had a sensitivity of 93% and specificity of 96% in detecting pleural effusions [22]. A meta-analysis from 2011 concluded that ultrasound had a higher sensitivity (88% v. 52%) and comparable specificity (99% v. 100%) to chest radiography for the diagnosis of pneumothorax [2]. A meta-analysis from 2014 found that ultrasound had a sensitivity of 94.1% and specificity of 92.4% in detecting acute cardiogenic pulmonary edema in dyspneic ED patients [23]. Finally, a meta-analysis from 2015 found that ultrasound had a sensitivity of 95% and specificity of 90% in detecting adult pneumonia [3]. In the same analysis, chest radiography was found to have a sensitivity of 77% and specificity of 91%. Few studies have examined differences in outcomes when POCUS is used as a diagnostic tool. A recent prospective study published in 2016 used POCUS as a supplement to the Advanced Cardiac Life Support (ACLS) protocol for patients with cardiac arrest [24]. The study was not randomized and all patients received ultrasounds. However, they found that POCUS was able to quickly identify reversible causes of cardiac arrest leading to subsequent appropriate interventions, such as pericardiocentesis for pericardial effusion, and tPA administration for pulmonary embolism.

Other advantages of POCUS include its ease of use at the bedside, the lack of ionizing radiation, and real-time results. This contrasts with the time requirements of chest radiography, which is an average of 36 minutes in the ED [25]. This figure is smaller in acute situations, as a 2014 retrospective study analyzing the door-to-CT time of ED patients receiving tPA for ischemic stroke found that those patients receiving chest radiographs had an increase in door-to-CT time of 13 minutes compared to those not receiving chest radiographs [26]. Even so, the utility of chest radiography may well be supplanted in the future. A prospective, interventional study published in 2016 proposed to eliminate chest and pelvic radiographs in hemodynamically stable patients and replace them entirely with the extended Focused Assessment with Sonography for Trauma (eFAST) protocol [27]. They found that patients receiving radiographs (most of whom received both chest and pelvic radiographs) stayed, on average, 13 minutes longer in the trauma bay [28].

While this study did not quantify the time required to perform POCUS, it is the authors’ experience that each component of the protocol takes less than one minute to perform in most conditions, and the results are immediate. In adverse conditions, the protocol may take fifteen minutes or more depending upon operator experience, interruptions from other healthcare providers, the patient’s ability to cooperate, and the patient’s body habitus. Fortunately, the RADiUS protocol is similar to the eFAST protocol, which is in common use. The protocols share similar probe positioning for the evaluation of pneumothorax, the hepatorenal recess, the splenorenal recess, and the heart. Emergency physicians trained in the eFAST have already learned much of the RADiUS protocol [27].

The use of a medical student is a novel approach to evaluate the sensitivity of POCUS protocols to user experience, and this study is one example of the results that novice ultrasound users can achieve. While there was no quantitative assessment of the medical student’s performance of POCUS technique, the archived images allowed for adequate interpretation by the supervising physician. The medical student interpreted 54 out of 59 protocols correctly (92%), which suggests that ultrasound interpretation in a clinical setting is feasible even for medical students. While there are no direct comparisons available, the accuracy of interpretation by the medical student falls roughly in line with previous studies on POCUS education. A group of 25 medical students underwent five hours of training on the Focused Assessment with Sonography in Trauma (FAST) protocol and scored an average of 86% on an objective structured clinical examination (OSCE), which included a FAST scan on a human volunteer [29]. Another group of six medical students and six residents were found to have 93% accuracy in the interpretation of POCUS simulations on the Abdominal and Cardiothoracic Evaluation by Sonography (ACES) protocol after focused training [30].

This study was subject to several limitations. First, due to limited study resources, inter-operator variability could not be assessed because one medical student performed all ultrasounds. Second, there was only one emergency attending physician who reviewed all the scans. Third, while studies were performed when the primary team was away from the bedside, there was no protocol for blinding the primary ED team to the POCUS results. The primary ED team therefore had access to the POCUS results if they chose to interview the medical student after the POCUS was performed. While unlikely, ED physician access to POCUS results could have introduced an incorporation bias affecting the discharge diagnosis. Even if this were the case, chest radiograph results would influence discharge diagnosis more strongly since they were not performed and interpreted by a medical student. Thus, the net incorporation bias would favor chest radiography. In addition to the patient, the primary ED team also had to be consented before performing the study. This added the possibility that ED teams could refuse or emphasize consent to select for certain types of patients. Fourth, the small sample size (59 patients) in comparison to the large number of diagnostic categories that were being evaluated (11 clinical patterns) made achieving statistical significance nearly impossible. In addition, generating a single set of sensitivity and specificity values from 11 diagnostic categories reduced the reliability and interpretability of these metrics compared with a study using a single diagnostic category. Fifth, the timing of the POCUS was not quantitatively compared to that of chest radiography, so temporal advantages could not be assessed in this study. Future studies should consider evaluating the modified RADiUS protocol in fewer categories and among multiple providers.

## Conclusions

Point-of-care ultrasound using the modified RADiUS protocol was compared with chest radiography in patients with undifferentiated respiratory and chest complaints. The sensitivity and specificity of POCUS was not significantly different from chest radiography. A medical student was able to perform the modified RADiUS protocol and interpret with a high level of accuracy. The modified RADiUS protocol could become a valuable tool in ED physician practice. However, further studies must validate its diagnostic value more robustly.
